# An Approach to Reinforce Multipath TCP with Path-Aware Information

**DOI:** 10.3390/s19030476

**Published:** 2019-01-24

**Authors:** Kien Nguyen, Mirza Golam Kibria, Kentaro Ishizu, Fumihide Kojima, Hiroo Sekiya

**Affiliations:** 1 Graduate School of Engineering, Chiba University, 1-33, Yayoi-cho, Inage-ku, Chiba-shi 263-8522, Chiba, Japan; sekiya@faculty.chiba-u.jp; 2 Wireless Systems Laboratory, National Institute of Information and Communications Technology, 3-4 Hikarinooka, Yokosuka 239-0847, Kanagawa, Japan; mirza.kibria@uni.lu (M.G.K.); ishidu@nict.go.jp (K.I.); f-kojima@nict.go.jp (F.K.)

**Keywords:** MPTCP, reinforce, path awareness

## Abstract

Multipath TCP (MPTCP), which enables the use of multiple wireless links (e.g., Wi-Fi and LTE) for data transmissions, is an excellent technology for evolving multi-homing devices in mobile wireless networks. This paper explores concepts and feasibility of realizing MPTCP with path awareness (PA), in which the path-aware information is leveraged to reinforce the MPTCP transmissions. In particular, when aware of a network situation, a device can facilitate a mechanism that dynamically shifts the MPTCP traffic to a single path and vice versa. As a result, MPTCP with PA could solve the significant problem of negative aggregation benefit when the MPTCP throughput over divergent paths is worse than the best one of single-path TCP. We illustrate the feasibility of the proposed concept through our new implementation of a so-called MPTCP-LA (i.e., MPTCP with Loss Awareness). MPTCP-LA keeps the aggregation benefits non-negative by temporarily switching an MPTCP transmission on a path to a standby condition when the on-device observed loss reaches a threshold. We extensively evaluate MPTCP-LA in comparison to the standard MPTCP in an emulated environment. The results show that MPTCP-LA has better performance regarding enhancing throughput and saving networking resources.

## 1. Introduction

Mobile devices and Internet access have become very important for human lives in recent years. Additionally, the popularity of Wi-Fi and the ubiquitous deployments of cellular networks have changed the behavior of Internet users. Instead of using wired connections, the Internet users prefer to access the Internet via wireless links (e.g., either Wi-Fi or LTE on their mobile devices). With various emerging applications such as real-time video streaming, there is a need to concurrently harness the resources of both Wi-Fi and LTE networks, for bandwidth or resilience enhancement. Along with the evolution to the fifth generation of the mobile wireless network (i.e., 5G), Multipath TCP (MPTCP) is one of the most successful technologies for realizing the concurrency. MPTCP is, in fact, a transport layer protocol that has been standardized by the Internet Engineering Task Force (IETF) [1]. MPTCP attracts interests from various device makers and vendors. We have seen MPTCP implementations on popular mobile platforms (e.g., Android [2], Apple iOSs [3]), as well as, real deployments [4].

MPTCP naturally evolves on existing networking infrastructures and applications without any modification. In operation, MPTCP on an end of communication separates application traffic into several TCP flows (i.e., subflows), each of which traverses over a wireless link (e.g., Wi-Fi and LTE). MPTCP on another end consequently aggregates the subflows, inherently improves the availability and fault tolerance in comparison to the conventional TCP. More importantly, MPTCP is expected to improve the overall throughput of the application by design. However, the throughput improvement does not always hold, MPTCP may deteriorate the aggregate throughput in heterogeneous wireless environments. The problem (i.e., negative aggregation benefit) has been confirmed by real experiments, in which the MPTCP throughput is sometimes smaller than the best one of single-path TCP [5]. If the mobile device is aware of such situation, it should switch to TCP instead of MPTCP. Besides, there are other exemplary use cases where the MPTCP/TCP switch is useful. For example, the MPTCP-capable device insists on using Wi-Fi, LTE only due to cost, security reason, respectively. Therefore, to reinforce MPTCP, it is necessary to devise a mechanism that can provide the adaptive transition between MPTCP and TCP in an on-demand manner.

This paper analyzes the feasibility of realizing the adaptive mechanism that decouples with path-aware information. The conceptual idea is that when the device is situationally cognizant, it will switch the continuous MPTCP transmission to TCP or vice versa. As a proof of concept, we propose MPTCP with loss awareness (i.e., MPTCP-LA), which aims to bypass the problem of negative aggregation benefit in a lossy wireless environment. We affirm that the problem appears by experimental study with the MPTCP standard in an emulated network, where the loss conditions have varied. The experiments also discover a threshold of lost packets on each path, which leads to the degradation of aggregation throughput. We have implemented MPTCP-LA on top of the standard MPTCP and compared the two MPTCPs. MPTCP-LA can temporarily stop an MPTCP subflow when the associated path’s lost packets reach a predetermined threshold. The comparative results show that MPTCP-LA has better overall performance than MPTCP in the lossy environment. In dynamic loss scenarios, MPTCP-LA efficiently switches back-and-forth between MPTCP and TCP, hence enhance the throughput and path use.

This work is in line with a current trend in the communication network, in which exploiting path information is for the communication quality ’s enhancement. Recently, IETF has started a Path-Aware Networking Research Group to investigate the feasibility and efficiency of path awareness communication at the transport and application layers [6]. Moreover, the efforts in spurring cellular network information to mobile devices let the devices become more aware of the network [7] (i.e., similar to the Wi-Fi information). There have been a few related works in improving multipath transmissions with path awareness [8,9]. They are however simulation- or theory-based. Regarding the practical approach, the work in [10] proposes a method that temporarily mutes a wireless link and shifts traffic to the other, but for energy efficiency. The most related work to ours is [11] in which the authors have introduced a switch mechanism for MPTCP on a device with two Wi-Fi links (i.e., MPTCP-MA). MPTCP-MA initializes the switch depending on aware information of signal strength, which is also determined by experiments. However, MPTCP-MA requires modifications on the standard MPTCP. Different to the others, we aim to reinforce MPTCP with the path-aware following an approach that maintains not only performance but also compatibility with the standard MPTCP.

The remainder of this paper is organized as follows. Section 2 presents the background information and related works. Section 3 introduces our approach and MPTCP-LA. Section 4 describes the evaluation methodology and results. Finally, Section 5 includes the conclusion and future works.

## 2. Background

### 2.1. Wireless Links on Mobile Devices

The Wi-Fi technology has been prevalent in the current era. Wi-Fi has been on many electric devices for a wide range of applications. On mobile devices such as phones or tablets, the default Internet connection is generally the Wi-Fi link. Additionally, there are continuous efforts in advancing Wi-Fi standards, each of which (e.g., IEEE 802.11 a/b/g/n/ac/ad/ax) will be available for commercial use within a few years. Accordingly, the achievable throughput of Wi-Fi link is significantly increased (from 11 Mbps in IEEE 11b to up to 300 Mbps in IEEE 802.11n or multi-Gbps in IEEE 802.11ad). For Internet access, the connection via a Wi-Fi link generally has higher throughput, shorter delay, but causes more lost packets in comparison to the one via the cellular link. However, that common observation does not hold longer due to the transformation of the mobile network in recent years (e.g., third generation (3G) to the fourth generation (4G/LTE)). In a 4G network, the theoretical peak speed for services is about 100 Mbps in high mobility environments, and 1 Gbps in low mobility environments. The cellular network performance is even expected to be significantly higher in the next generation of mobile wireless networks (i.e., 5G). Additionally, the real measurement also confirms that fact as in [5], where the authors show the cellular throughput is as similar as the Wi-Fi’s one. Therefore, the difference between the two technologies is only related to the range of signal coverage (i.e., LTE has broader coverage than Wi-Fi). We reflect the difference by the values of latency via each path in our evaluation.

### 2.2. Multipath Transmission Control Protocol

#### 2.2.1. Overview of MPTCP Operation

MPTCP is an extension of the conventional TCP for enabling communication over multiple paths (i.e., routes) each of which is formed by a pair of IP addresses between two ends. Similar to TCP, MPTCP initializes a connection in a three-way handshake method of using SYN, SYN/ACK, ACK for the first subflow (e.g., via a Wi-Fi link). Different to TCP, the first SYN of MPTCP additionally contains an MP_CAPABLE option, an authentication key, which is for checking MPTCP-capability and adding later subflows, respectively. If the SYN receiver is capable of MPTCP, it responds by sending an SYN/ACK with the MP_CAPABLE option and its authentication key. After that, the transmission of an ACK packet with the two keys is accomplished to complete the initialization of the first subflow. When the mobile device has an extra IP address (for example associated with an LTE link), MPTCP will begin a new handshake for the second subflow. The SYN has an MP_JOIN option beside the key. If the handshake is successful, MPTCP will recognize the new subflow. The subflow is then attached to the MPTCP connection by sending another ACK. Consequently, the device concurrently achieves data transmissions through two routes to a destination.

In MPTCP, there are several support working modes which determine several paths for data transmission. MPTCP could use either subset or total available IP pairs between two ends of the communication. However, the operation modes necessarily require pre-configuration without any dynamic or flexibility of switching between them.

#### 2.2.2. MPTCP Structure

**Figure 1:**
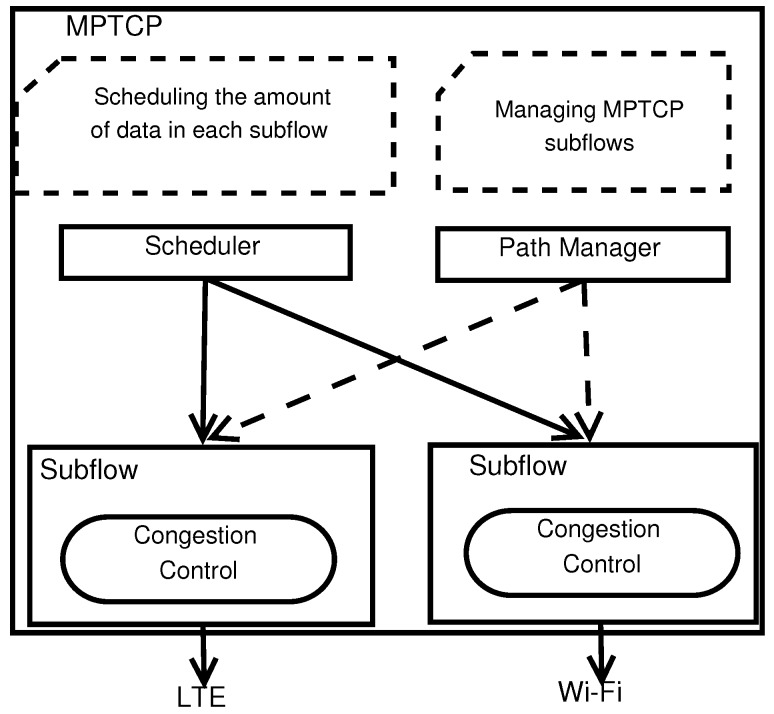
Software Component in MPTCP.

The MPTCP software contains three main components, which are Scheduler, Path Manager, and Congestion Controls as depicted in Figure 1. The details of each module is described below.

- **Scheduler:** After an MPTCP connection creates and attaches multiple subflows, it will divide a certain amount of data to be transmitted on a subflow. That duty is in charge of the MPTCP Scheduler which operates a scheduling algorithm. The default one selects the subflow with the smallest round-trip time (RTT) among multiple subflows, for which there is available space for congestion window size achieving data transmission.
- **Path Manager:** In the conventional TCP, there is only one route (path) between two ends of communication, path management at transport layer is unnecessary. However, in MPTCP, the Path Manager module is needed to manage the multiple paths efficiently. Each path (i.e., equivalent to a subflow) is identified by a pair of the ID of the source IP address and the ID of the destination IP address. The default Path Manager of MPTCP does not add subflow unless there is a request from the other.
- **Congestion control:** Similar to TCP, MPTCP also use congestion control algorithms for congestion avoidance. MPTCP could use the congestion control mechanisms of TCP. However, each subflow will be an independent TCP flow in that case. That may cause an unexpected performance reduction. Therefore, there are several the MPTCP congestion controls, which decouples the congested states of all subflows (e.g., *coupled* [12], *olia* [13], *wvegas* [14], *balia* [15]). The *balia* has been proven to be more effective than the others in [15].

### 2.3. Related Works

There are recently a lot of interests on MPTCP adoption on mobile wireless networks. MPTCP has been proven to be suitable with not only the current but future wireless networks. For example, MPTCP is efficient on mobile devices with Wi-Fi/3G [10,16], Wi-Fi/Wi-Fi [11], virtual Wi-Fi/virtual Wi-Fi [17], Wi-Fi/WiGig [18], 5G networks [19], and future software defined wireless networks [20,21], etc. Besides that, there are ongoing efforts in spreading MPTCP to popular platforms not only Android, iOS but also Linux [22], FreeBSD [23], etc. MPTCP enables concurrent transmissions over multiple wireless links for the throughput and resilience improvements. Due to the dynamic nature of mobile wireless, the MPTCP’s efficiency on mobile devices needs to be carefully investigated and optimized. Accordingly, there have been many related researches in recent years.

In [16], the authors show the capability of soft switch-over between Wi-Fi/3G using MPTCP for throughput and energy efficiency. In [24], the authors conduct the measurement of MPTCP performance in the wild. The throughput results match the design criteria of MPTCP, which is the MPTCP throughput is always not lower than the best single-path TCP. However, a similar measurement in [5] shows a different result. The throughput of MPTCP is worse than the best TCP in several scenarios, especially the ones with loss. Therefore, MPTCP needs to be improved to bypass the negative performance. In [10], an MPTCP scheduler is proposed but for an energy efficient purpose. The scheduler probes and collect information from all interfaces in a short period, and then switches to the most efficient one. MPTCP-MA in [11] is proposed for a scenario of Wi-Fi/Wi-Fi networks. MPTCP-MA arranges the wireless link usage to recover throughput quickly and reduce packet losses in a path failure resulting from a sudden Wi-Fi disconnection. MPTCP-MA is aware of the Wi-Fi signal strength, hence it is similar to our approach. However, MPTCP-MA requires modifications of the standard MPTCP. On the other hand, we aim to improve MPTCP without any modification of the standard. We exploit the concept of path-aware information, which is visible on the device. Regarding the path aware communication, there is a newly established IETF working group (i.e., Path-Aware Networking Research Group) aiming to standardize path awareness communication at the transport and application layers [6]. Moreover, there have been a few related works in improving MPTCP with path awareness [8,9], in which the delay awareness has been addressed. However, the authors do not directly target MPTCP, and they use the simulation or theory-based methods. Our approach, which is specifically designated for MPTCP, contains a real implementation to prove the feasibility. Our preliminary work has been published in [25]. This paper adds the detail explanation of the concept, the feasible realization, and evaluation results.

## 3. An Approach to Reinforce MPTCP with Path Awareness

### 3.1. Motivation

MPTCP has efficiently worked on a wide range of wireless technologies, but it still has disadvantages. One of the widely known issues is that MPTCP cannot always achieve the benefit of throughput aggregation, especially in the scenarios with divergent paths. Even in its canonical use case of LTE/Wi-Fi network, the MPTCP throughput is lower than the best TCP when the path characteristics (e.g., RTTs, loss conditions, etc.) are much different. The throughput degradation also appears in another essential use case of MPTCP in an evolving Wi-Fi network with WiGig and Wi-Fi links, when the WiGig link (i.e., with a directional antenna) is available. However, the reason is mainly due to the difference between the maximum transmission unit (MTU) values on the Wi-Fi and WiGig links [18]. The leading cause of the degradation is that an MPTCP connection tries to establish and use all subflows by default. The MPTCP subflow uses two types of sequence (i.e., at the subflow and connection levels) to guarantee the correct order of packet transmissions. Therefore, the earlier arrived packets of a subflow need to buffer and wait for correctly resequencing. Thus, if an event that causes the waiting timeout (e.g., loss, long transmission time) occurs, the influence extends not only to the relevant subflow but also to the whole MPTCP communication. It is hence expected to have a mechanism that shifts ongoing MPTCP traffic to a single path to solve the problem. Moreover, when the condition that caused the degradation becomes acceptable, MPTCP could be dynamically reverted to the full functionality.

It is worthy to note that there are other use cases where the shift is useful. For example, in a public place, a user may want to use only the LTE link for the sake of security. Another user may insist on the Wi-Fi network since the monthly traffic reaches the cap. In those examples, it is not difficult for the devices to be aware of the wireless networks’ parameters.

### 3.2. Feasibility Analysis

In this section, assuming the awareness of path information, we analyze the feasibility and challenges in realizing the expected function without modification of the standard MPTCP.

In the standard, an MPTCP subflow can be configured to operate in either the normal or the backup mode. By default, all the initialized subflows of an MPTCP connection are in the former mode. The subflows are then used to transmit data (i.e., managing TCP sequence at the subflow level). When an MPTCP subflow is in the latter, there is no data transmission on such subflow unless there is no other normal-mode subflow. To set the two modes, we have to process on a per-interface basis. When set, all subflows that use the interface share the same mode.

In an end-to-end MPTCP communication, if an interface is configured to be in a backup mode at one end, a notification with an MP_PRIO option is sent to the other end for the recognition of the backup subflow. To completely set the backup mode of subflow on both ends, it takes about a half of Round-Trip Time (RTT) of the associated path. Theoretically, we could leverage the operation modes of MPTCP to realize the expected mechanism that dynamically configures the subflow operation. However, the primary challenge is that the mode configuration is either manual or preconfigured. Additionally, the configuration tasks are out of the MPTCP kernel (i.e., the userspace). Hence, they may cause delay and unstable even though they operate successfully.

To bypass the challenge, we aim to realize all the configuration tasks within the MPTCP kernel. In particular, we newly integrate a path-aware scheduling algorithm in the MPTCP scheduler. The algorithm frequently checks the path/network information, which is visible on the device, following predetermined criteria. When the device is aware of the situation, in which the MPTCP performance is worse or better. The algorithm will trigger the subflow configuration, which will let the subflow be in the backup or normal mode, respectively. Accordingly, it will realize the switch of MPTCP transmission back-and-forth for the good performance. The triggering point may rely on any aware information from transport to physical layers. The switch mechanism is hence a critical component that could be shared by different path-aware criteria. The uniqueness of our mechanism includes using on-device information and without modification of the standard MPTCP. We will show the feasibility through the implementation of the MPTCP with loss awareness (i.e., MPTCP-LA) in the next section, where we use the loss information as the trigger.

### 3.3. MPTCP with Loss Awareness

This section presents MPTCP-LA, which uses the loss-aware information on devices to achieve the switch mechanism, hence solves the problem of negative aggregation benefit. Since MPTCP works at the transport layer, it generalizes all types of loss. Moreover, only the congestion control algorithm is in charge of handling with lost packets. The behavior of congestion control is considerably slow since it has to wait until there is an indication signal from another end or after a timeout value. In such cases, the loss information (e.g., from wireless drivers, probing network, or management information [26]) are likely visible on the device. Therefore, if it can be aware of the throughput degradation on a lossy path, it should let MPTCP stop the transmission on that path by activating the proposed switch mechanism.

To be aware of the aggregation throughput of MPTCP, we rely on the aggregation benefit function [27], which has been used to investigate the efficiency of multipath communications. We denote the function as Ben(M) specified as follows.

(1) $$Ben\left(M\right)=\left\{\begin{array}{cc} \frac{G-C_{max}}{\sum_{i=1}^{n}C_{i}-C_{max}}, & \mathrm{if}\;G\geq C_{max}\\ \frac{G-C_{max}}{C_{max}}, & \mathrm{if}\;G<C_{max} \end{array}\right.$$

where *M* be a multipath scenario, with *n* paths, and $C_{i}$ is the capacity (i.e., TCP throughput) of the path *i*, $C_{max}$ is the highest capacity among all paths, *G* is the measured MPTCP throughput. Ben(M) provides a convenient way to observe the aggregation benefit since all the function values are in the range $\left[-1,1\right]$. The value range not only makes the performance in different scenarios comparable but also reveals the impact of MPTCP. Specifically, if Ben(M)≥0 or $G\geq C_{max}$, MPTCP has a comparable throughput performance (i.e., non-negative benefit) than the best TCP one over a single path. It suggests we should use MPTCP. On the other hand, TCP should be used if Ben(M) is negative. We adopt the experimental study method to quantify the values of Ben(M) in the lossy scenarios which are in the next section. The evaluation results will provide the aware information of the threshold values of lost packets that cause the negativity of aggregation benefit.

In MPTCP-LA, the switch algorithm between normal to backup mode is as follows. For each subflow, a threshold value of lost packets (denoted as γ), which is the loss-aware information, is preconfigured. During an MPTCP session, after every *N* packet transmission, for each subflow the number of transmitted packets *mptcp_tx_cnt* and the number of lost packets *mptcp_loss_cnt* are tracked. The numbers are used to calculate the real lost rate and compare with the threshold.

$$\frac{mptcp\_loss\_cnt}{mptcp\_tx\_cnt}\geq \gamma$$

when the above condition holds, MPTCP-LA achieves the switch mechanism if the subflow is not the only one in the normal mode. In our implementation, the switching toggles a newly added flag to configure the backup mode. MPTCP-LA also handles the case of multiple subflows with exceeded loss threshold. In such case, the algorithm lets the subflow with the higher loss percentage be in the backup mode. All the steps are inside the MPTCP kernel (i.e., in MPTCP Scheduler), they hence are fast and accurate. The switch action from the backup to normal mode also uses the toggled flag. However, it cannot be achieved fully inside the MPTCP kernel since there is no transmission on the backup subflow. To facilitate that, MPTCP-LA uses the kernel information (e.g., Linux kernel) on the device.

To realize our expected mechanism, we have initially modified the data structure of MPTCP subflow to have the counted numbers of packets. Moreover, we have newly declared a data structure in the MPTCP scheduler that includes variables. The first important one is the so-called *look_over_subflows*, which takes the MPTCP socket as an input argument. It confirms the entire subflows which contained by the socket and sets the flag mentioned above (i.e., *mptcp_change_flag*) of the subflow with the backup mode to 1. The second variable is used to interface with the system (i.e., *set_syscl_values*), which takes the first argument as of the number of received packets in the proc file system that is necessary for computing the packet loss rate of a subflow. The second argument is the loss threshold setting of backup mode for the subflow, that is also read from the proc file system. The third one is a pointer function, which is called at the beginning of the data transmission function of the MPTCP layer (i.e., the *mptcp_write_xmit()* function in *mtpcp_output.c*). The variables are controlled by a new variable in the MPTCP control block (i.e., *mptcp_cb*). Within the scheduler, we can manage the awareness comparison and switching an ongoing subflow to a backup one. However, to do the reverse method (backup to normal mode), we have to add the *cancel_backup_mode* for the scheduler newly. It takes the information from the proc system, then switches and updates the subflows’ status.

## 4. Evaluation

### 4.1. Setup

**Figure 2:**
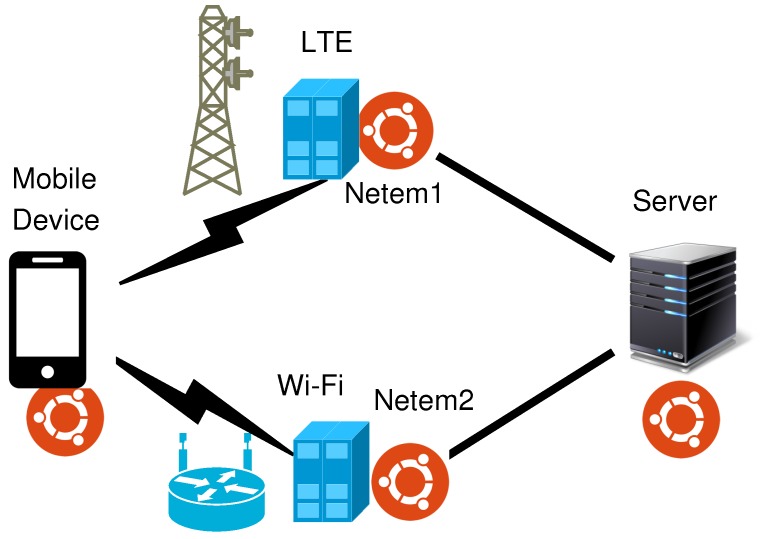
Evaluation scenario.

We set up an emulated network in an indoor environment to evaluate our proposal. The network emulates a client-server communication in a heterogeneous wireless scenario with Wi-Fi and LTE links as shown in Figure 2. In the figure, an MPTCP-capable device can communicate with an application server via the two paths associated with the wireless links. The device and the server connect to two Linux machines to form the network. On each Linux machine, the tool named *tc* is used to configure the path settings. The baseline value of delay on two paths is inherited from the previous works [5]. In each experiment, the jitter values are randomly selected in a range aiming to capture the dynamic of total delay values. The delay, jitter, and the set of bandwidth values are shown in Table 1.

We have implemented MPTCP-LA from MPTCP kernel release 0.90 [22], which are installed on the client and server. They can switch between the standard MPTCP and MPTCP-LA before an experiment. In our evaluations, we use the congestion control balia. To support the operation of MPTCP-LA, we develop a loss emulation module that runs in the device’s kernel. Each interface on the device has a new configuration file under the Linux’s proc that contains a value of loss probability. The module will drop packets following the appropriate probability. To reduce possible interferences, we disable route caching, buffer auto-tuning on the Linux machines. In each run, *iperf3* generates the TCP flows between the device and server.

### 4.2. Investigate the Aggregation Benefit in Lossy Environment

In this investigation, we find the correlation between the aggregation benefit and the loss conditions by experimental study. The study is motivated by the work in [28], where the same method is applied to characterize the MPTCP performance. First, we benchmark the capability of each path (i.e., TCP throughput) in normal and loss conditions. We experiment each set of parameters ten times and record the maximum values of TCP throughput among the measurements. Those values are filled in the aggregation function (i.e., $C_{i}$, $C_{max}$) after each experiment with the standard MPTCP. In the MPTCP evaluation, we vary the loss and bandwidth parameters on the Wi-Fi while there is no loss on the LTE link and vice versa. All the possible combinations in those scenarios have been evaluated. After each run, we collect the measured throughput of MPTCP and calculate the value of aggregation benefit.

**Figure 3:**
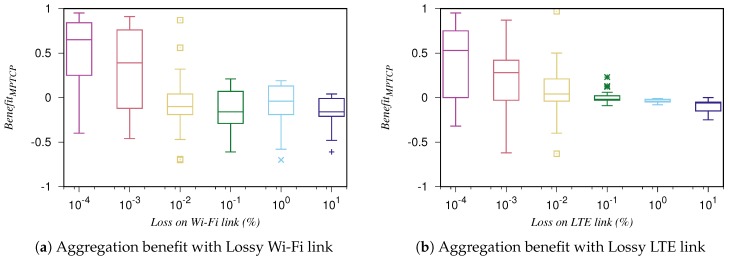
The aggregation benefit of MPTCP in different loss conditions.

We plot all the calculated values of aggregation benefit in Figure 3, in which Figure 3a,b shows the values associated with the scenarios of lossy Wi-Fi link and LTE link, respectively. In each sub-figure, the x-axis shows the log scale of loss values while the y-axis presents the values of benefit function. The boxplots show the average, minimum, maximum values and the distribution of values. We also plot the outliers in the figures. Except for the outliers, we observe that the aggregation benefit decreases when the loss ratio on a path increases. Figure 3a shows when the loss of Wi-Fi path equals to or larger than 0.01%, the benefit function tends to be zero or negative. In such scenarios, even though MPTCP shifts the traffic to the less lossy link (i.e., LTE), the MPTCP throughput is worse than the TCP throughput on the LTE path. Therefore, it is expected to apply the switching mechanism (from the normal to backup mode) for subflow on the Wi-Fi link, when the loss reaches a threshold of 0.1%. In the case of LTE path, the same trend could be observed; however, the loss threshold is 1% as shown in Figure 3b. That is because the delay on LTE path is larger than the one on the Wi-Fi path.

### 4.3. Performance Comparison between MPTCP and MPTCP-LA

This section shows the performance comparison between MPTCP-LA and the standard MPTCP. Initially, we repeat the same experiments in the previous section for MPTCP-LA. We then consider the case of dynamic loss condition.

#### 4.3.1. Improve throughput with the Loss-Aware Mechanism

**Figure 4:**
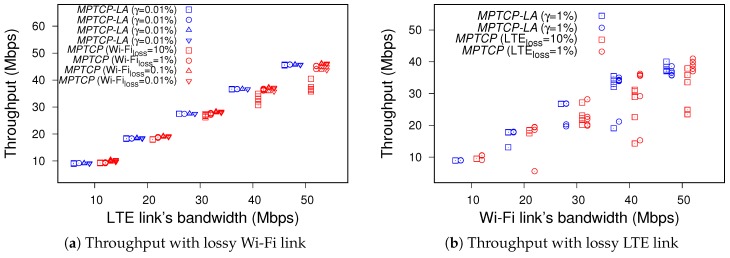
Throughput comparison between the default MPTCP and MPTCP-LA.

MPTCP-LA on the device is configured with the threshold values each of which is associated with an interface as found in the previous section. The Wi-Fi and LTE interface is assigned the threshold γ of 0.01% and 1%, respectively. The comparison of throughput of the two MPTCPs is shown in Figure 4.

Figure 4a shows the results in different scenarios of lossy Wi-Fi link. In the figure, the same shape of point shows the same link conditions, while the MPTCP’s throughput is in red; and MPTCP-LA’s one is in blue. The first observation is that the Wi-Fi link causes dropped packets, the total MPTCP throughput seemingly equals the capacity of the LTE path. When the bandwidth of Wi-Fi link is small, the MPTCPs have comparable throughput. Note that, MPTCP-LA still has benefit in that case since the switching mechanism could enable the power and radio resource saving on the LTE interface (i.e., from a switching moment to an end of experiment). When the bandwidth value of Wi-Fi path is larger than 20 Mbps, it is clear that the MPTCP throughput is much lower than the MPTCP-LA’s. That is because the retransmissions on the Wi-Fi subflow cause the retransmissions of the connection level. MPTCP-LA can avoid that case, hence the throughput is significantly improved. We can draw the same conclusion about MPTCP-LA from Figure 4b, where the LTE link experiences loss over the preconfigured threshold.

#### 4.3.2. Evaluation in Dynamic Loss Environment

This evaluation aims to show the adaptive behavior of MPTCP-LA in an environment with dynamic loss conditions. We choose the average measured throughput as the metric describes the ability to react to varying loss conditions. Moreover, the path use is chosen as an indication of the efficient usage of networking resource. We use the same network scenario as in Figure 2. The delay and jitter values of LTE and Wi-Fi paths are also similar, the two paths’ bandwidth is 50 Mbps. We evaluate MPTCP-LA and MPTCP in following the following 90-second scenario. In the first 30 s, both the LTE and Wi-Fi paths are configured in a normal condition (i.e., without loss). Then Wi-Fi path experiences 5% of the lost packets, which is initiated at the 30th second. After that, the loss of Wi-Fi path returns to zero around the 60th second. The experiment is repeated ten times for each MPTCP.

**Figure 5:**
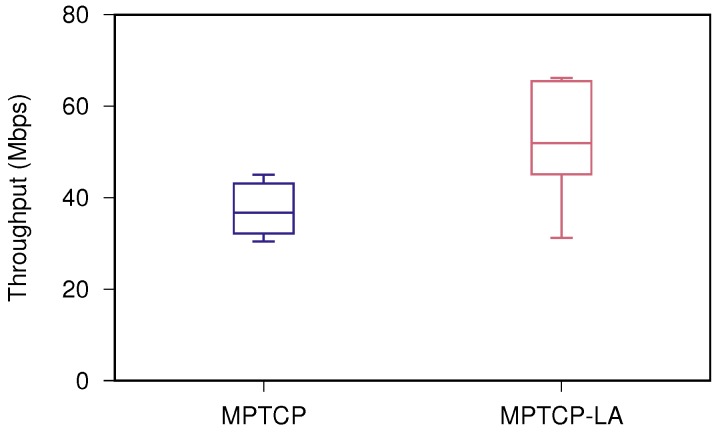
Throughput comparison in dynamic loss condition.

Figure 5 shows a comparison of the average throughput between two MPTCPs. In the figure, each box plot shows the distribution, average, minimum, maximum values of throughput of all runs. We have the same observation as in the previous throughput evaluation. MPTCP-LA with the loss-aware mechanism outperforms the standard MPTCP. Not only that, MPTCP seems to rarely have the aggregation benefit since most of its throughput values are less than the path bandwidth.

**Figure 6:**
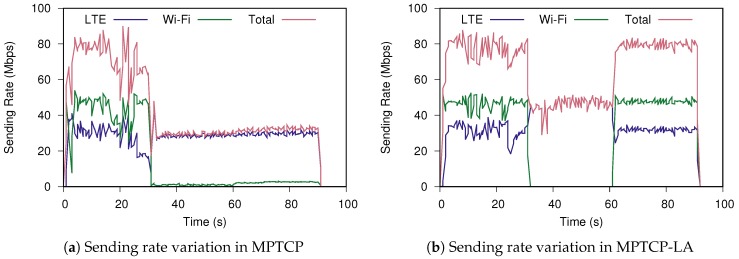
Comparing path use between MPTCP and MPTCP-LA.

Regarding the usage of networking resource, we show the variation of sending rate in one typical run of each MPTCP as an indicator of path use in Figure 6. We use the tool bwm-ng [29] to track the sending rat variation of MPTCP and MPTCP-LA shown in Figure 6a,b, respectively. In this case, the standard MPTCP is able to switch most part of the traffic from the lossy path to the good path as in Figure 6a. However, it has no reaction after the Wi-Fi becomes loss-free although two paths are still active. On the other hand, Figure 6b shows MPTCP-LA well adapts to the loss variation. During 30th–60th second, there is no loss in MPTCP-LA’s transmission hence it has higher rates than MPTCP. The resource of the backup path can be used for another purpose (e.g., turn off for energy saving). Therefore, we can conclude MPTCP-LA uses the networking resource more efficiency than MPTCP.

## 5. Conclusions and Future Works

This paper introduces an approach to reinforce MPTCP with the path-aware on-device information. In particular, the MPTCP-capable device with LTE/Wi-Fi links can automatically switch between the multipath and single-path communication when it is aware of the path information. We have proven the approach’s feasibility through the implementation of MPTCP-LA (i.e., MPTCP with loss awareness), in which the switch mechanism is activated following the loss-aware information. Specifically, MPTCP-LA temporarily stops or resume an MPTCP transmission over wireless link when the observed value of lost packets is over or below a threshold, respectively. Consequently, MPTCP-LA can solve the MPTCP problem of negative aggregation benefit in the lossy environment. We have evaluated the MPTCP-LA performance in comparison to the standard MPTCP. The evaluation results show that the MPTCP-LA significantly achieves better performance concerning throughput and resource saving in the lossy environments.

In the future, we plan to extend the proposed switch mechanism with other awareness information (e.g., RSSI) and compare with the other state-of-the-art such as [11].

## Figures and Tables

**Table 1:** Path parameter.

	Delay (ms)	Jitter (ms)	Bandwidth (Mbps)
Wi-Fi	10	[0, 5]	(10, 20, 30, 40, 50)
LTE	75	[0, 35]	(10, 20, 30, 40, 50)
